# Freshwater Sponges as a Neglected Reservoir of Bacterial Biodiversity

**DOI:** 10.3390/microorganisms12010025

**Published:** 2023-12-22

**Authors:** Angelina Lo Giudice, Carmen Rizzo

**Affiliations:** 1 Institute of Polar Sciences, National Research Council (CNR.ISP), Spianata S. Raineri 86, 98122 Messina, Italy; carmen.rizzo@szn.it; 2 Zoological Station “Anton Dohrn”, Department of Ecosustainable Marine Biotechnology, Villa Pace, Contrada Porticatello, 98168 Messina, Italy

**Keywords:** freshwater sponges, bacterial communities, bacterial isolates, bacterial symbionts, biotechnological potential, biodiversity

## Abstract

Freshwater sponges (Spongillida: Demospongiae), including more than 240 described species, are globally distributed in continental waters (except for Antarctica), where they cover both natural and artificial surfaces. However, fragmentary studies have targeted their microbiome, making it difficult to test hypotheses about sponge-microbe specificity and metabolic relationships, along with the environmental factors playing key roles in structuring the associated microbial communities. To date, particular attention has been paid to sponges (family *Lubomirskiidae*) that are endemic to Lake Baikal. Few other freshwater sponge species (e.g., *Ephydatia* spp., *Eunapius* spp., and *Spongilla lacustris*), from lakes and rivers spanning from Europe to South and North America, have been targeted for microbiological studies. Representatives of the phyla *Proteobacteria*, *Bacteroidetes*, and *Actinobacteria* largely predominated, and high differences were reported between the microbiome of freshwater and marine sponges. Several bacterial strains isolated from freshwater sponges can produce bioactive compounds, mainly showing antibiotic activities, with potential application in biotechnology. Understanding the roles played by sponge microbiomes in freshwater ecosystems is still in its infancy and has yet to be clarified to disentangle the ecological and evolutionary significance of these largely under-investigated microbial communities. This review was aimed at providing the main available information on the composition and biotechnological potential of prokaryotic communities associated with healthy freshwater sponges, as a neglected component of the global sponge microbiome, to stimulate researchers interested in the field.

## 1. Introduction

Among the oldest extant multicellular animals, sponges (Phylum Porifera) are sessile benthic filter-feeding macroinvertebrates commonly found in aquatic habitats worldwide. All sponges pump high amounts of water through their specialized aquiferous system. A collagenous extracellular matrix (called mesohyl) covers the system of sponge canals, filling the space between the ectoderm (i.e., the outer cell layer) and the pinacoderm (composed of cells lining the inner canals). Choanocytes (i.e., flagellated cells), organized in choanocyte chambers, are responsible for the flux of water from the exterior, through pores called ostia, to the internal canal system. Food particles suspended in pumped water are transferred into the mesohyl and then digested by specialized amoeboid cells, called archaeocytes, which are embedded in the three-dimensional extracellular matrix of the sponge [1].

The ecological, evolutionary, and microbiological significance of sponges is unquestioned [2]. Filtering large volumes of water, sponges play crucial roles in the coupling of benthic and pelagic ecosystems due to the recycling of both inorganic and organic nutrients (e.g., silicon, carbon, and nitrogen) [3,4]. Thanks to their complex three-dimensional structures, sponges provide habitat and refuge from predators for several species (including microinvertebrates, microalgae, and microeukaryotes), thus enhancing ecosystem biodiversity and functioning [5]. Sponges can be colonized by prokaryotic symbiotic assemblages, with most of them that are species-specific and phylogenetically diverse, strongly differing from those inhabiting the surrounding environment. It has been demonstrated that the body weight of some marine sponge species (namely, high microbial abundance sponges, HMA) can be composed of 40% of microorganisms [6,7], reaching an abundance of 10^8^–10^10^ microbes g^−1^ sponge wet weight, contrary to low microbial abundance (LMA) sponges that host around 10^5^–10^6^ microbial cells g^−1^ [8,9]. Symbiotic microbes are highly involved in the ecology and physiology of their host (for instance, its development, behavior, and resistance to disease); for this reason, the sponge host and the associated microbiota are often altogether described as a “holobiont”, i.e., a unique biological entity with genetic features (i.e., the hologenome) that are also contributed by the microorganisms [10].

Freshwater sponges (Spongillida: Demospongiae) include more than 240 described species (organized into six families and 47 genera) [11]. In inland waters, both lentic and lotic systems (e.g., lakes, ponds, rivers, streams, man-made reservoirs, and garden ponds) can be found covering both natural (e.g., stones, wood, macrophytes, or invertebrates) and anthropic (e.g., glass, metal) substrata assuming branched, encrusting, or cloddy bulk shapes. They largely varied in color and dimensions, ranging from a few mm to 1.50 m in size [12]. A greenish appearance is frequent in light-exposed habitats, due to the occurrence of algal or cyanobacterial symbionts supplying photosynthates to the sponge and intervening in the protection against UV radiation [1,13]. Freshwater sponges are highly adapted to drastic changes characterizing environmental conditions of freshwater systems (e.g., thermal fluctuations, fluctuating water levels, long-lasting desiccation, temporary anoxic conditions, and pollutant occurrence) thanks to modifications at the molecular, physiological, and structural level [11]. For instance, freshwater sponges can produce gemmules, encapsulating undifferentiated cells into silica structures, to survive under winter conditions [11]. 

**Figure 1:**
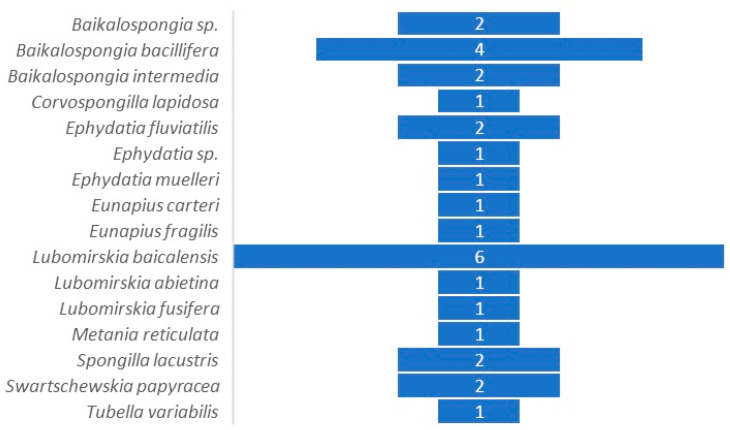
Number of studies targeting bacterial communities associated with freshwater sponges.

To date, several studies have examined microorganisms associated with marine Porifera, which include more than 95% of known sponge species. However, even if freshwater sponges offer similar ecosystem functions in freshwater habitats [2], sporadic and fragmentary studies have targeted their microbiology, making it difficult to test hypotheses about sponge-microbe specificity (even in unconnected freshwater systems) and metabolic relationships, along with the environmental factors playing key roles in structuring the associated microbiome [2]. Particular attention has been paid to the sponge species *Lubomirskia baicalensis*, including diseased individuals, which are endemic to Lake Baikal [14,15,16,17,18]. Conversely, fragmentary data are reported on the microbiota associated with a few other sponge species (e.g., *Corvospongilla lapidosa*, *Ephydatia fluviatilis*, *E. muelleri*, *Eunapius carteri*, *Spongilla lacustris*, and *Tubella variabilis*), from other freshwater systems (Figure 1). 

Most studies applied culture-dependent methods for the characterization of bacterial isolates, including their potential as producers of biotechnological relevant molecules [16,17,18,19], whereas high-resolution taxonomic data on the whole communities, obtained by next-generation sequencing technologies, remain still limited (e.g., [2,20,21,22,23]). Overall, the prokaryotic communities are generally characterized by the predominance of *Proteobacteria*, *Bacteroidetes*, and *Actinobacteria* and highly differ from those determined in marine sponges [21]. Sugden et al. [2] reported for the first time the comparison between sponge-associated communities and the surrounding environments (e.g., water and biofilm). This latter aspect is of major importance to establish if discrepancies between prokaryotic communities in sponges and water can match the differences that may be observed between benthic and planktonic communities rather than the functional significance of host-microbe specificity [2].

The sponge immune system constitutes a first line of defense against transient microbiota, including the production of biomolecules that recognize conserved microbial structures [24]. Moreover, bacterial symbionts are probably involved in the sponge defense strategies to prevent predation, e.g., by fishes and mollusks, and impede colonization by unwanted organisms (e.g., microbial biofilms and fouling) through the production of antagonistic metabolites [25]. Different from marine sponges, our current knowledge on this aspect in inland water sponges remains poorly investigated. It is noteworthy that more than 100 distinct bioactive compounds, that can be useful for humans, have been detected in freshwater sponges and some of them might originate from symbiotic bacteria [26,27]. 

This review was aimed at providing the main available information on the composition and biotechnological potential of prokaryotic communities associated with healthy freshwater sponges, as a neglected component of the global sponge microbiome. 

## 2. Prokaryotic Communities Associated with Freshwater Sponges: Main Findings by Culture-Independent Approaches 

In the following sections, our current knowledge on the composition, and possibly functions, of bacterial communities associated with freshwater sponges, as determined by culture-independent methods (including clone library construction and next-generation sequencing, NGS), is showcased *per* sponge species (listed in Table 1). 

### 2.1. Ephydatia *spp.*

*E. fluviatilis* (Linnaeus, 1759) is a sponge species common to continental water bodies (both lotic and lentic) in the northern hemisphere, particularly in Central Europe. The molecular diversity and composition of bacteria in *E. fluviatilis*, from the artificial lake Vinkeveense Plassen (Utrecht, The Netherlands), was explored for the first time by Costa et al. [28], who suggested a selective process by the host organism to choose associated microbes. By applying the polymerase chain reaction-denaturing gradient gel electrophoresis (PCR-DGGE) fingerprints, the authors observed that sponge- and water-derived bacterial communities differed in composition at the phylum level, with no or negligible overlaps between the *E. fluviatilis* and water-derived phylotypes within bacterial taxa. For instance, *Actinobacteria*, *Proteobacteria*, and *Bacteroidetes* dominated in the freshwater clone library, whereas *Proteobacteria*, *Planctomycetes*, *Actinobacteria*, *Bacteroidetes*, *Chlamydiae,* and *Verrucomicrobiota* were more abundant in water. The co-dominance of other bacterial phyla, such as the candidate phylum TM7, was also reported. Interestingly, a distinct and less diversified actinobacterial community was observed in *E. fluviatilis*, with a sharp selection of an uncultured actinobacterial phylotype in the order *Acidimicrobiales* [28]. Within *Gammaproteobacteria*, a previously unsuspected complexity of *E. fluviatilis*-associated *Pseudomonas* assemblages was revealed by the search for the *gacA* gene as a phylogenetic marker alternative to conventional 16S rRNA marker gene, which instead failed to reflect the multiplicity of these organisms in their sponge host [25]. Within *Alphaproteobacteria*, two clusters recovered from *E. fluviatilis* were related (at about 93–94% sequence similarity) to sequences previously obtained from *S. lacustris* [1] and *L. baicalensis* [29]. The same was observed for a sponge-specific cluster within *Bacteroidetes*, which was previously reported within *L. baicalensis* community [29], and for a *Polynucleobacter* sp. cluster that was similar (>96%) to a *Polynucleobacter necessarius* from *S. lacustris* [1]. According to the authors, whether these phylotypes represent freshwater sponge-specific lineages, thus supporting the hypothesis on the existence of co-evolutionary relationships between hosts and symbionts, remains to be demonstrated.

**Table 1:** Studies targeting freshwater sponge prokaryotic communities by culture-independent methods.

Sponge Species	Freshwater Sampling Site	Reference(s)
*Baikalospongia* sp.	Lake Baikal (Russia)	[30,31]
*Baikalospongia bacillifera* (Dybowsky, 1880)	Lake Baikal (Russia)	[22]
*Baikalospongia intermedia* (Dybowski, 1880)	Lake Baikal (Russia)	[32]
*Corvospongilla lapidosa* (Annandale, 1908)	Talegaon Dabhade and Pashan (India)	[20]
*Ephydatia fluviatilis* (Linnaeus, 1759)	Vinkeveense Plassen Lake (The Netherlands)	[25,28]
*Ephydatia muelleri* (Lieberkühn, 1856)	Six locations in the Northern Hemisphere	[22]
	Sooke, Cowichan, and Nanaimo Rivers (Canada)	[2]
*Eunapius carteri* (Bowerbank, 1863)	Talegaon Dabhade and Pashan (India)	[20]
*Lubomirskia baicalensis* (Pallas, 1776)	Lake Baikal (Russia)	[15,30,31,32,33]
*L. abietina* (Swartschewsky, 1901)	Lake Baikal (Russia)	[33]
*Spongilla lacustris* (Linnaeus, 1759)	Lake Staffelsee (Germany)	[1]
	Pichlinger See Lake (Upper Austria)	[23]
*Swartschewskia papyracea* (Dybowsky, 1880)	Lake Baikal (Russia)	[32]
*Tubella variabilis* (Bonetto and Ezcurra de Drago, 1973)	Artificial channel (Brazil)	[21]

*E. muelleri* (Lieberkühn, 1856) is a cosmopolitan and globally abundant sponge species. Its chromosomal-level genome has been well annotated [22]. This species is frequently studied for some physiological, biological, and genetic sponge aspects, especially in relation to animal evolution [34,35]. Some sets of *E. muelleri* genes have been proven to be involved in the establishment and maintenance of symbiotic interactions [36]. To date, this sponge has been the subject of two papers dealing with its associated microbiomes, analyzed by culture-independent approaches [2,22]. Kenny et al. [22] reported a microbiome composition of *E. muelleri* (from six different freshwater locations of the Northern Hemisphere) that was comparable to that observed in most marine demosponges, with *Proteobacteria* and *Bacteroidetes* as predominant microbial components. However, differently from marine sponges, *E. muelleri* hosted a large fraction of *Betaproteobacteriales* (absent in marine sponges). Overall, microbial content differed by geographic location. For instance, samples from the Sooke Reservoir had a higher abundance of *Firmicutes* and *Campylobacteria*, whereas those from Maine were characterized by a moderate abundance of *Cyanobacteria*. Notably, despite that sponge individuals were collected from distant sites (with potentially different ecological features), the authors detected few amplicon sequence variants (ASVs) that were shared among all samples, occurring at different relative percentages, including *Burkholderiaceae* (order *Betaproteobacteriales*) and *Ferruginibacter* (order *Chitinophagales*). These findings represented a baseline for the study of species-specific patterns of host–microbe association in freshwater systems at a broader scale. Later, Sugden et al. [2] applied the 16S rRNA gene amplicon sequencing complemented with shotgun metagenomics to describe the microbiome of *E. muelleri* from the Sooke, Nanaimo, and Cowichan Rivers on Vancouver Island (British Columbia, Canada), also in relation to ambient water and adjacent biofilm. The authors demonstrated that *E. muelleri* core microbiome, besides being different from those of the surrounding environment, included *Comamonas*, *Diaphorobacter*, *Methylotenera*, *Rhodoferax*, unclassified *Rhodospirillales*, and *Sediminibacterium*, with the predominance of *Sediminibacterium*, *Comamonas* and *Rhodospirillales* (overall accounting for 58%). Notably, the dominant *Sediminibacterium* (as amplicon sequence variant, ASV) in sponge communities differed from the dominant *Sediminibacterium* ASV in both water and biofilm samples. Conversely, the most abundant ASV for *Comamonas* and *Rhodospirillales* were consistent across all tested matrices and rivers. Interestingly, most abundant *Sediminibacterium* and *Rhodospirillales* sequences were closely related (sequence identities ≥ 98.42%) to uncultured bacteria from different sponge species from a number of freshwater systems [1,14,15,16,17,18,20,28], supporting the hypothesis that evolutionarily conserved sponge-bacteria associations may exist also in freshwater environments. Although the microbiome composition was largely conserved among different rivers, as was previously observed by Kenny et al. [22], sponges were distinguished by their origin based on a few microbial features. In detail, higher relative abundances were determined for *Pseudarcicella*, *Flavobacterium*, and *Fluviicola* in Sooke sponges; *Polynucleobacter* in Nanaimo sponges; and *Sediminibacterium* and *Parcubacteria* in Cowichan sponges. This finding again suggested that other environmental or host-specific variables may affect the observed geographic variations. Results obtained by Sugden et al. [2] differed from those previously obtained by Kenny et al. [22] for *E. muelleri* sponges and gemmules from upstream of the Sooke River, with the microbiome that better resembled those of analyzed biofilms. Finally, shotgun metagenomes and metagenome-assembled genomes revealed that the microbiome of *E. muelleri* can show some compositional and functional similarities (e.g., defense-related proteins and genes for vitamin B12 production) with prokaryotic communities associated with marine sponges [2].

### 2.2. Eunapius carteri and Corvospongilla lapidosa 

The microbiota of the globular sponge *E. carteri* (Bowerbank, 1863) and the encrusting sponge *C. lapidosa* (Annandale, 1908) (from the permanent freshwater lake located at Talegaon Dabhade and Pashan, India, respectively) was explored for the first time using next generation sequencing (NGS) technology by Gaikward et al. [20]. The authors also analyzed lake water for microbiota composition. Overall, 14 bacterial phyla were detected, with more than 2900 and 980 OTUs (higher than those generally reported for marine sponges) that were obtained from *C. lapidosa* and *E. carteri*, respectively. The two sponge-associated microbial communities strongly differed: *E. carteri* community was dominated by *Firmicutes*, followed by *Proteobacteria* and *Cyanobacteria*; *C. lapidosa* community was characterized by a higher abundance of *Proteobacteria*, followed by *Planctomycetes*, *Cyanobacteria*, and *Actinobacteria*. The authors suggested that this finding probably was dependent on the differences encountered in the measured environmental variables, highlighting the crucial role played by the habitat in structuring the associated microbial communities. Among the main phyla, *Cyanobacteria* are considered minor components of the bacterial communities associated with freshwater sponges. However, Gaikward et al. [20] determined a high abundance of *Cyanobacteria* in both sponges (comparable with those reported for marine sponges). In particular, *Synechococcus* sequences were abundantly present only in *E. carteri*, whereas *Planktothrix* and *Planktothricoides* were abundant in *C. lapidosa*. Nevertheless, the role played by *Cyanobacteria* in their association with sponges (as a food or symbiont) remains to be elucidated. The study also revealed that the structure of both microbial communities was significantly different from their respective water samples (i.e., dominant *Proteobacteria*, followed by *Bacteroidetes*, *Actinobacteria,* and *Cyanobacteria* in the water next to *E. carteri*; dominant *Actinobacteria*, followed by *Bacteroidetes*, *Proteobacteria*, *Planctomycetes,* and *Cyanobacteria* in the water next to *C. lapidosa*), implying that the host genetic factors could be also involved in the species-specific association. Notably, *Nitrospirae*, *Chloroflexi*, *Chlamydiae,* and *Acidobacteria* occurred only within the *C. lapidosa* bacterial community. Conversely, *Firmicutes* were retrieved only in association with *E. carteri*, with more than 50% of their sequences being affiliated with the genus *Clostridium* (absent in the surrounding water). Gaikward et al. [20] supposed that the high abundance of *Clostridium* might be due to the ability of members in this genus to utilize some complex molecules (such as proteoglycans, glycoproteins, collagen, and spongin) that are present in the sponge extracellular matrix. However, the authors deserved to verify if, as observed in marine sponges, the occurrence of *Clostridium* spp. might derive from the exposition to environmental stresses (e.g., toxic chemicals). Finally, based on the detection of several bacterial lineages (belonging to *Firmicutes*, *Actinobacteria*, *Proteobacteria*, and *Planctomycetes*) that are known to produce compounds of biotechnological relevance, the authors encouraged the isolation and characterization of microbes from freshwater sponges to be screened for their biotechnological potentialities.

### 2.3. Lubomirskiidae Family from the Lake Baikal

At least 14 species of freshwater sponges are endemic to Lake Baikal (Russia) and belong to the Lubomirskiidae family. The Lubomirskiidae include species such as Lubomirskia baicalensis, *Baikalospongia bacillifera*, *B. intermedia*, *B. martinsoni*, and *Swartschewskia papyracea*. Most studies on the symbiotic bacterial communities have been focused on *L. baicalensis* and *Baikalospongia* spp., as reported below.

*L. baicalensis* (Pallas, 1776) is the dominant endemic sponge species of Lake Baikal. It lives in symbiosis with a green okadaic acid-producing dinoflagellate (closely related to related to *Gymnodinium sanguineum*), involved in the host survival in the iced lake in winter [37]. The diversity of the microbial community associated with the branching *L. baicalensis*, together with that of an encrusting *Baikalospongia* sp., was evaluated by NGS for the first time by Gladkikh et al. [30], highlighting a complexity in diversity level that was comparable to that estimated for marine sponges. Overall, in *L. baicalensis* 6873 (out of 7071) 16S rRNA gene sequences belonged to the bacteria domain (with 2935 of them that were unique). A total of 426 identified phylotypes grouped in four main bacterial phyla, i.e., *Bacteroidetes* (48% of total sequences), *Proteobacteria* (28%), *Actinobacteria* (14.7%), and *Planctomycetes* (7.3%), followed at lower abundance by *Verrucomicrobiota*, *Nitrospirae*, OD1, and *Chloroflexi*. Sequences not affiliated with known phyla were also observed. These findings were in line with previous observations previously made by applying clone library approach, allowing the detection of *Actinobacteria* (37%), *Proteobacteria* (*Alpha*- and *Betaproteobacteria*, 22 and 13%, respectively), *Verrucomicrobia* (11%), *Bacteroidetes* (7.5%), *Cyanobacteria* (7.5%), and *Nitrospira* (2%), even if they occurred at different relative percentages [15] As was observed for *Baikalospongia* sp. from the same study by Gladkikh et al. [30], *Sediminibacterium* affiliates predominated, accounting for 40.4% of the total community. Among *Proteobacteria*, *Polynucleobacter necessarius* (family *Burkholderiaceae*, *Betaproteobacteria*) constituted 22.7% of the total community, in accordance with results on clones from *E. fluviatilis* [28] and *Baikalospongia* sp. [30]. Additionally, *Pedomicrobium australicum* (family *Hyphomicrobiaceae*; *Alphaproteobacteria*) represented 4.9% of the bacterial community associated with *L. baicalensis*. The third most abundant phylum, i.e., *Actinobacteria*, was exclusively composed of the common planktonic species *Planktophila limnetica*. Overall, 6817 (out of 7042) 16S rRNA gene sequences from *Baikalospongia* sp. belonged to the bacteria domain (with 2601 of them that were unique) [30]. A total of 428 phylotypes were identified and grouped into four main bacterial phyla, i.e., *Bacteroidetes* (52.3% of total sequences), *Proteobacteria* (28.7%), *Actinobacteria* (9.5%), and *Planctomycetes* (7.7%), followed at lower abundance by *Verrucomicrobiota*, OD1 and *Chloroflexi*. Sequences not affiliated with known phyla were also observed. Within *Bacteroidetes*, the genus *Sediminibacterium* accounted for 47.0% of the total community. Among *Proteobacteria*, *Polynucleobacter necessarius* (family *Burkholderiaceae*, *Betaproteobacteria*) constituted 12.0% of the total community, in accordance with previous results on clones from *E. fluviatilis* [28]. The third most abundant phylum, i.e., *Actinobacteria*, was exclusively composed of the common planktonic species *Planktophila limnetica*. Finally, *Planctomycetes* were mostly represented by *Phycisphaera mikurensi* (7.2% of the total community). A high sequence homology (95–97%) was observed between the two analyzed sponge species, namely *Baikalospongia* sp. and *L. baicalensis*, and the bacterioplankton from the same site of collection in Lake Baikal. Furthermore, sponge communities shared 75 common OTUs at the level of species (97% homology), with over 50% of the bacterial phylotypes of each sponge being unique, and 55% of the planktonic phylotypes that were not retrieved in associations with sponges.

Jung et al. [31] tested a new isolation method from *L. baicalensis* and *Baicalospongia* sp., comparing the obtained results with those from pyrosequencing. Overall, 13,964 and 7496 reads were analyzed from *L. baicalensis* and *Baicalospongia* sp., respectively. *Actinobacteria*, *Alphaproteobacteria*, *Firmicutes*, *Betaproteobacteria*, *Cyanobacteria*, *Planctomycetes*, and *Verrucomicrobia* were retrieved in both sponges (except for *Verrucomicrobia* in *L. baicalensis*). *Cyanobacteria* predominate within both associated bacterial communities, accounting for 70–78% of total reads, whereas *Gammaproteobacteria* abundance was negligible (<1%). 

Seo et al. [32] analyzed the bacterial diversity of the Baikalian sponges *L. baicalensis*, *B. intermedia* Dybowsky, 1880 and *S. papyracea* (Dybowsky, 1880) (the latter reported for the first time) by pyrosequencing highlighted some differences in their bacterial community composition, suggesting that bacterial symbiont community composition was mainly affected by environmental conditions or gradients across water depths in Lake Baikal. A total of 7496, 13,654, and 4941 reads were retrieved from *B. intermedia*, *L. baicalensis,* and *S. papyracea*, respectively. Overall, *Cyanobacteria* predominated (range 42–78%), followed by *Proteobacteria*. In particular, *Cyanobacteria* were affiliated with the orders *Synechococcales*, *Chroococcales* (found only in *L. baicalensis* and *S. papyracea*) and *Nostocales* (retrieved only in *B. intermedia* and *S. papyracea*). The abundance of the genus *Prochlorococcus* was in the range of 40–74% in all sponge species, with *Prochlorococcus marinus* surprisingly, being generally found in the marine environment, occurring only in *S. papyracea*. *B. intermedia* and *L. baicalensis* hosted a highly similar bacterial community, differing from that associated with *S. papyracea* (showing a higher bacterial diversity). *Actinobacteria* abundance was three times higher in *S. papyracea* (13.4%) than *B. intermedia* and *L. baicalensis* (3.87 and 3.46%, respectively), while *Acidobacteria* and *Gemmatimonadetes* occurred only in *S. papyracea*. Ten reads in the orders *Micrococcales*, *Bacteroidales*, *Verrucomicrobiales,* and *Solirubrobacterales* occurred only in *S. papyracea*. The authors hypothesized that the bacterial community associated with *S. papyracea* was sponge-specific and adapted for specific environmental conditions (such as lower light levels or higher pressure than the other two sponge species).

More recently, Kenny et al. [33] compared the genomic content and microbiome of *L. baicalensis* and *B. bacillifera*, in addition to *L. abietina* Swartschewsky, 1901, to gain knowledge on their molecular evolution to adapt to Lake Baikal’s unique environment. Overall, the dataset included a small number of bacterial sequences, representing a subsample of complete bacterial diversity. According to the authors, bacterial genomes were not inordinately represented in the dataset, indicating that they were not well assembled. However, the obtained results were generally consistent with previous data on other freshwater sponge species (e.g., [1,20,28,32]). Briefly, in *L. baicalensis* Actinobacterial sequences (1589 annotated contigs) were predominant, followed by *Proteobacteria* (1087 contigs) and *Candidatus Tectomicrobia* sp. (191 contigs). *Firmicutes*, *Bacteroidetes*, *Cyanobacteria*, *Verrucomicrobia*, and *Chlamydiae* (in order of decreasing occurrence) were less represented. In *L. abietina* 407 and 203 proteobacterial and *Candidatus Tectomicrobia* contigs were retrieved, followed by *Actinobacteria*, *Firmicutes*, *Bacteroidetes*, *Cyanobacteria*, *Verrucomicrobia*, and *Chloroflexi*. *Chlamydiae* contigs were only eight. In *B. bacillifera*, Kenny et al. [33] observed the predominance of *Proteobacteria* (392 contigs), followed by *Candidatus* Tectomicrobia (157 contigs) and, at a lesser extent, by *Bacteroidetes*, *Firmicutes*, *Actinobacteria*, *Cyanobacteria*, and *Verrucomicrobia*. Only nine contigs of the obligately intracellular *Chlamydiae* occurred. Overall, the most common bacterial symbiont in the three sponge species was *Candidatus Entotheonella geminae*. According to the authors, this symbiotic bacterium may be necessary for the survival of the host playing roles that are useful in freshwater in general, and Lake Baikal in particular, such as the protection against heavy metals, supply of energy in anaerobic conditions, and support in CO_2_ fixation.

### 2.4. Spongilla lacustris 

*S. lacustris* (Linneus, 1759) is a widespread (and seldom fast-growing, especially in Central Europe) sponge species generally inhabiting temperate regions of the Northern Hemisphere. Although it is a very effective biological filter, being able to remove and consume large amounts of particulate matter in water, *S. lacustris* growth mainly depends on photosynthetic products supplied by the intracellular symbiotic algae [38,39]. To the best of our knowledge, to date, the bacterial community of *S. lacustris* has been described in depth only in a couple of papers by applying culture-independent methods [1,23]. Gernert et al. [1] constructed 16S rRNA gene libraries from both sponge tissues and Lake Staffelsee (Germany) water. By the application of the restriction fragment length polymorphism (RFLP) analysis performed on 9190 freshwater sponge-derived clones, 45 clones (clustering in six major restriction patterns) were selected for sequencing. The authors observed the predominance of *Alphaproteobacteria* and *Actinobacteria* (51 and 36%, respectively), followed by *Betaproteobacteria* and *Chloroflexi* (5% each). The sponge-associated bacterial community was highly similar to that observed for Lake Staffelsee. However, two alphaproteobacterial sequences appeared to be novel and freshwater sponge-specific.

More recently, Graffius et al. [23] investigated the microbiome of *S. lacustris* from Lake Pichlinger See (Upper Austria) using a combined approach, which coupled the analysis of bacterial isolates (including their genome analysis) with those of 16S rRNA gene amplicons and metagenomes from sponge tissue DNA extracts. As it was determined by 16S rRNA gene sequencing, a wide fraction of ASVs was not classified at the genus level, being strongly related to uncultured and unclassified *Bacteroidetes*, *Alphaproteobacteria*, *Gammaproteobacteria*, and *Betaproteobacteria*, highlighting a high degree of phylogenetic novelty in the *S. lacustris* microbiome. Metagenome binning, resulting in 20 metagenome-assembled genomes (MAGs) did not reveal the occurrence of sponge-related taxa. Instead, MAGs are mostly related to metagenomes of freshwater and marine origin, as well as hot springs, hypersaline lakes, and pathogenic isolates from shrimps and diseased fish.

### 2.5. Tubella variabilis 

*T. variabilis* (Bonetto and Ezcurra de Drago, 1973) is an encrusting thin sponge species. It is beige and green in color and fragile to moderately soft in consistency [40]. Laport et al. [21] compared the microbial community structure of *T. variabilis* specimens from an artificial channel, providing water to fish farm tanks (Recife, Pernambuco, Brazil), with that of the surrounding freshwater. Bacterial diversity was higher in sponges than in water. Overall, *Proteobacteria* dominated both communities, followed in their relative abundances by *Bacteroidetes*, *Acidobacteriota*, *Verrucomicrobiota*, and *Cyanobacteria*. However, *Alphaproteobacteria* were enriched in *T. variabilis*, whereas *Betaproteobacteria* dominated in freshwater. Among *Bacteroidetes*, which were more abundant in the sponge than in water, *Cytophagia*-related sequences were a hundred times higher in *T. variabilis* than in freshwater. To gain further insight, the authors tested the difference in the relative abundance of the most abundant OTUs (representing 64% of the total community). Among them, 20 strongly differed between the two habitats (sponge and water). For instance, the methanotrophic genus *Methylosinus* (among *Alphaproteobacteria*) and family Cytophagacea (among *Bacteroidetes*) were enriched in *T. variabilis*. Conversely, the genera *Methylocaldum*, *Sulfuricurvum* and genus C39 (order *Rhodocyclales*), and family *Comamonadacea* were more abundant in freshwater. Laport et al. [21] also compared the richness (measured by number of OTUs) and structure of the microbial community of *T. variabilis* with those of two other sponges, namely *E. carteri* and *C. lapidosa* from Indian freshwaters [20], along with 32 different sponges of marine origin. *T. variabilis* and *C. lapidosa* harbored the richest communities. Notably, freshwater and marine sponges are markedly grouped in separated clusters by nMDS. Since freshwater sponges were collected from geographically distant areas (i.e., India and Brazil) (collected in Brazil), the authors suggested that water salinity might be a possible main factor structuring the microbial community associated with sponges. 

**Figure 2:**
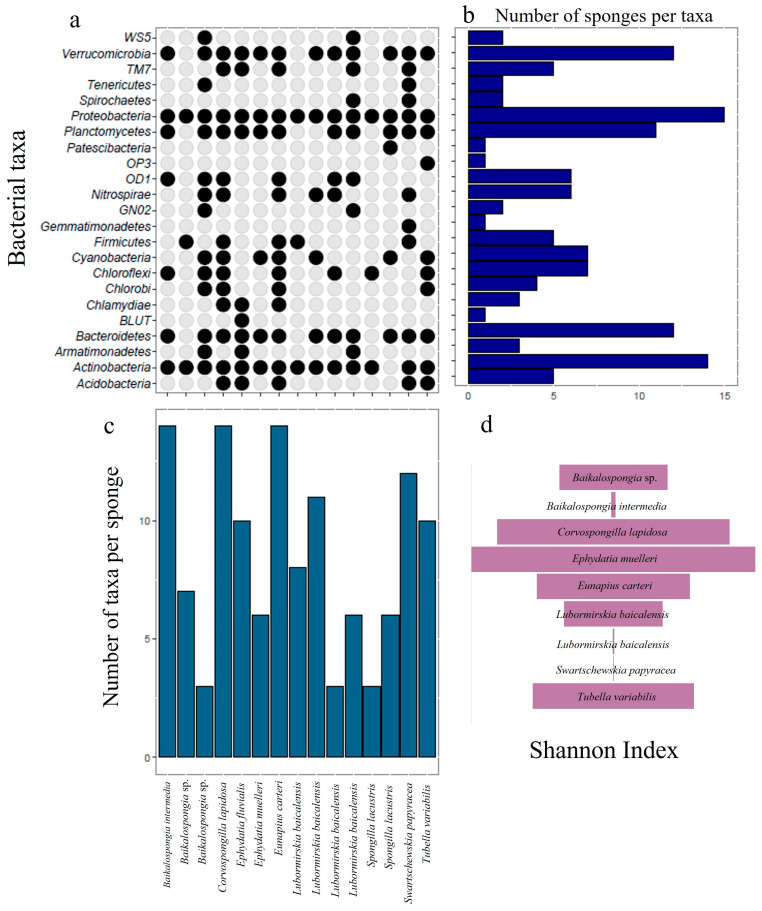
Graphical representation of bacterial taxa detected in freshwater sponges from different sites. Presence/Absence matrix of bacterial taxa detected in different freshwater sponges (**a**); number of sponge specimens in which each taxon was detected (**b**); number of taxa detected in each sponge species/specimen (**c**): *Baikalospongia intermedia* [32], *Baikalospongia* sp. [30], *Baikalospongia* sp. [31], *Corvospongilla lapidosa* [20], *Ephydatia fluvialis* [25], *Ephydatia muelleri* [2], *Eunapius carteri* [20], *Lubormirskia baicalensis* sp. [30], *Lubormirskia baicalensis* [32], *Lubormirskia baicalensis* [31], *Lubormirskia baicalensis* [29], *Spongilla lacustris* [1], *Spongilla lacustris* [23], *Swartschewskia papyracea* [32], *Tubella variabilis* [21]; Shannon diversity index detected in bacterial communities associated with freshwater sponges (**d**): *Baikalospongia* sp. [30], *Baikalospongia intermedia* [32], *Corvospongilla lapidosa* [20], *Ephydatia muelleri* [2], *Eunapius carteri* [20], *Lubormirskia baicalensis* sp. [30], *Lubormirskia baicalensis* [32], *Swartschewskia papyracea* [32], *Tubella variabilis* [21]. *Please note that Shannon Index is reported only when available from literature*.

An overview of the freshwater sponge species investigated so far, and their geographical locations is given in Figure 2. It is a graphical representation of available data regarding the taxonomic composition of bacterial communities associated with freshwater sponges. Despite some bacterial taxa being overall shared between the various investigated sponge species, it is possible to appreciate the presence of some peculiar taxa in certain sponges, such as *Spirochaetes*, *Tenericutes,* and *Nitrospirae*.

## 3. Bacterial Isolates from Freshwater Sponges: Description and Biotechnological Potential

A number of bacterial strains have been isolated and described from freshwater sponges so far. In the following sections, current data on bacterial isolates, including the description of novel species, are reported *per* sponge species (cfr. Section 3.1), if dealing with the characterization of the cultivable bacterial fraction, or *per* biotechnological potential (cfr. Section 3.2), if dealing with isolates of biotechnological value. 

### 3.1. Characterization of the Cultivable Fraction of the Freshwater Sponges-Associated Bacterial Communities 

To date, the cultivable fraction of the bacterial communities associated with freshwater sponges has been analyzed for eight sponge species, spanning from Austria to Russia (Table 2).

#### 3.1.1. *Baikalospongia bacillifera*, *B. intermedia*, *Lubomirskia fusifera*, *L. baicalensis* and *Swartschewskia papyracea* from Lake Baikal

A total of 77 bacterial strains were isolated from the sponges *B. bacillifera* Dybowski, 1880 (58 isolates), *L. fusifera* Soukatschoff, 1895 (13 isolates), and *S. papyracea* (Dybowsky, 1880) (6 isolates) collected in Lake Baikal near the village of Bol’shie Koty [14]. Strains affiliated with the genera *Bacillus*, *Flavobacterium*, *Pseudomonas*, and *Acinetobacter* were isolated from all sponge species. *Micrococcus* and *Arthrobacter* occurred only in *L. fusifera* and *B. bacillifera*, whereas *Sarcina* was retrieved only in *S. papyracea*. However, some genera were also isolated from lake water, with *Pseudomonas* that prevailed in water, and *Bacillus*, *Micrococcus*, and *Sarcina* that were more abundant in sponges. Parfenova et al. [14] isolated psychrophilic bacteria from the sponges *L. baicalensis*, *B. bacillifera*, and *B. intermedia* Dybowsky, 1880 collected near Cape Berezovyi (Lake Baikal). *Streptomyces* and *Micromonospora* affiliates were probably permanent components of the bacterial communities. In particular, *Micromonospora* accounted for 68, 69, and 90% of all actinomycetes in *L. baicalensis*, *B. intermedia*, and *B. baicalensis*, respectively. Later on, Jung et al. [31] applied a novel method, called I-tip, for the isolation of bacteria from *L. baicalensis* and *Baicalospongia* sp. The method missed only two major phyla detected by pyrosequencing, as it allowed the isolation of 34 bacterial species from five major phyla (i.e., *Actinobacteria*, *Alphaproteobacteria*, *Betaproteobacteria*, *Firmicutes*, and *Gammaproteobacteria*). Among them, *Nocardia*, *Rhodococcus*, *Microbacterium*, *Brevundimonas*, *Sphingomonas*, *Acinetobacter*, and *Pseudomonas* representatives were isolated from both sponge species. Conversely, three major phyla (namely, *Betaproteobacteria*, *Firmicutes*, and *Gammaproteobacteria*), including 16 bacterial species, were obtained by standard cultivation method, failing the detection of some major phyla determined by pyrosequencing. However, the I-tip-derived culture collection did not include a high abundance of *Gammaproteobacteria*, such as the commonly isolated *Pseudomonas*. Conversely, *Actinobacteria* and *Alphaproteobacteria* were enriched by the I-tip method. The authors conclude that the I-tip method can better represent the natural bacterial diversity, narrowing the gap between cultivated and uncultivated species, at least in the case pf host-associated microbial communities.

#### 3.1.2. *Ephydatia* sp.

Based on the moderate relative abundance (14.4% of total bacterial abundance) of *Planctomycetes* reported by Costa et al. [28], Kohn et al. [41] enriched with N-acetyl-d-glucosamine (NAG) as sole carbon source samples of an *Ephydatia* sp. from Lake Constance (Germany), targeting bacteria from the phylum *Planctomycetes*. Following this approach, the authors isolated and first described a novel species, namely *Planctopirus ephydatiae* (strain spb1^T^). Contrary to chemotaxonomic measurements (including cellular fatty acid analysis) that showed similarities of strain spb1^T^ with the related species *P. limnophila* (DSM 3776T), the genomic analysis revealed that strain spb1T represented a yet unknown species of *Planctomycetes*.

#### 3.1.3. *Eunapius fragilis*

Two *E. fragilis* (Leidy, 1851) individuals were collected 1.5 km apart in the St. Lawrence River, North America [42]. A total of 851 isolates were obtained and identified as representatives of four major phyla commonly associated with sponges, i.e., *Proteobacteria* (including the genera *Yersinia*, *Rahnella*, *Enterobacter*, *Pseudomonas*, and *Delftia*), *Actinobacteria* (genera *Micromonospora*, *Verrucosispora*, and *Streptomyces*), *Bacteroidetes* (genus *Chryseobacterium*), and *Firmicutes* (genera *Bacillus* and *Paenibacillus*). The authors demonstrated that the two sponges shared many of the same genera but exhibited higher diversity at the species and possibly subspecies level. Isolates were screened for specialized metabolite production (see Section 3.2).

#### 3.1.4. *Spongilla lacustris*

Graffius et al. [23] identified 197 bacterial isolates from *S. lacustris* collected in Lake Pichlinger See (Upper Austria) by the 16S rRNA gene sequencing, with the purpose of testing their biotechnological potentialities (see the following section). The phylogenetic analysis revealed that isolates were representatives of Gram-negative and Gram-positive bacteria within 28 and 13 genera, respectively. The former were mostly affiliated with *Alphaproteobacteria* (mainly in the genera *Rhizobium* and *Brevundimonas*), followed by *Gammaproteobacteria* (mainly affiliated with *Pseudomonas* spp.) and, at a lesser extent, by few *Bacteroidetes* and *Verrucomicrobiota*. Gram-positive isolates were mainly affiliated with *Actinobacteria*, including the genera *Microbacterium*, *Frigoribacterium*, *Streptomyces*, and *Micrococcus*. *Bacillus* and *Exiguobacterium* members were among *Firmicutes* isolates.

#### 3.1.5. *Tubella variabilis*

By using five different culture media, Laport et al. [21] isolated 104 bacterial strains from *T. variabilis* (Bonetto and Ezcurra de Drago, 1973) (and an additional 59 isolates from freshwater) collected in an artificial channel receiving water from the da Prata River (Brazil). By the 16S rRNA gene sequencing, the strains were resolved in a total of 23 genera, with 12 of them that were isolated exclusively from the sponges. Most sponge-associated bacterial isolates were affiliated with *Proteobacteria* (namely *Alpha*-, *Beta*-, *Delta*-, *Gammaproteobacteria* classes). *Gammaproteobacteria* predominated in *T. variabilis* and were mainly represented by the family *Enterobacteriaceae*, with the genera *Klebsiella* and *Enterobacter*. The sole alphaproteobacterial isolate, belonging to the genus *Methylobacterium*, was isolated from a sponge sample. Among *Firmicutes*, which was the second most frequently isolated bacterial phylum, *Fictibacillus* and *Bacillus* were the predominant genera, with *Bacillus* that was not isolated from freshwater samples. *Actinobacteria* were also isolated only from sponges and were represented by a single isolate of the genera *Microbacterium*, *Micrococcus*, *Rhodococcus*, and *Streptomyces*. Finally, a unique *Bacteroidetes* isolate from *T. variabilis* was affiliated to the genus *Chryseobacterium*.

### 3.2. Biotechnological Relevant Bacteria from Freshwater Sponges

Recently, Clark et al. [42], combining MALDI-TOF mass spectrometry and the bioinformatics pipeline IDBac, analyzed both diversity and specialized molecule production of 692 bacterial strains isolated from *Eunapius fragilis*. Results highlighted the high biotechnological potential of bacteria isolates of freshwater sponges-associated bacteria. Notably, the specialized molecule production profiles varied at the bacterial species level and below, suggesting that in the search for novel bioactive compounds bacterial taxa should be analyzed on a case-by-case basis. To date, bacterial isolates from six freshwater sponge species have been targeted for their biotechnological potential as producers of molecules with antibiotic activities and enzymes (Table 3). Additional four sponge species have been targeted for the search of polyketide synthase (PKS) encoding genes within the whole microbial community (see below).

#### 3.2.1. Potential for the Production of Bioactive Compounds by Bacterial Isolates

According to Keller-Costa et al., [25] freshwater sponges constitute a reservoir of *Pseudomonas* spp. (among *Gammaproteobacteria*) of biotechnological value due to their antimicrobial activity. For instance, novel polyketide synthase (PKS) encoding genes (codifying for a number of bioactive compounds) were reported for a *Pseudomonas fluorescence* isolate (namely, strain 28Bb08) from *B. bacillifera* [43]. The low homology level between the obtained amino acid gene sequences and the sequences of known biologically active compound synthases suggested that strain 28Bb08 probably produced a far undescribed bioactive compound. Later, Keller-Costa et al. [25] addressed the diversity and in vitro antimicrobial activities of *Pseudomonas* spp. isolated from *E. fluviatilis*, demonstrating their antibacterial, antiprotozoan, and antioomycetal activities. Some strains were also able to inhibit the growth of basidiomycetal and ascomycetal pests. This finding suggests that *E. fluviatilis* is a promising source of underexplored *Pseudomonas* strains. Their diversified bioactivity spectrum might characterize sponge-associated pseudomonads, with relevance in their functionality within the holobiont. 

Axenov-Gribanov et al. [44] isolated and characterized actinobacterial strains from dominant benthic organisms’ communities of Lake Baikal, including *B. bacillifera*. Two isolates, namely *Streptomyces* sp. IB2014/01-2 and *Pseudonocardia* sp. IB2014/02-2, were screened for antibiotic activities against *Bacillus subtilis*, *Staphylococcus carnosus*, *Pseudomonas putida*, *E. coli*, and *Saccharomyces cerevisiae*. Both strains were able to inhibit the growth of *S. cerevisiae* and *E. coli*. *C. albicans,* and *S. carnosus* were inhibited by *Streptomyces* sp. IB2014/01-2 and *Pseudonocardia* sp. IB2014/02-2, respectively. The inhibitory activity varied based on the culture medium used for growth.

The 20.2% (21 out of 104) of bacterial isolates from *T. variabilis* showed inhibitory activity against *Staphylococcus aureus* [21]. Active bacteria were affiliated with the genera *Aquitalea* and *Chromobacterium* (both within *Betaproteobacteria*), *Dickeya* (within *Gammaproteobacteria*), and *Klebsiella* (within *Enterobacteriaceae*). 

Graffius et al. [23] selected 33 isolates (representing 31 bacterial genera) from *S. lacustris* collected in Lake Pichlinger See (Upper Austria) for genome sequencing. Overall, results revealed the occurrence of 306 secondary metabolite biosynthesis gene clusters (BGCs). According to the authors, although using different culture media for bacterial isolation, the cultivable fraction did not reflect the whole bacterial community (as it was determined by metagenome and 16S rRNA gene amplicon sequencing analyses; see the section above for *S. lacustris*), suggesting that isolates may be either low-abundance representatives of the *S. lacustris* associated bacterial community or transient bacteria. Two *Streptomyces* isolates (namely strains SL203 and SL294; *Actinobacteria*) showed the highest secondary metabolite biosynthesis potential, as they harbored 28 and 23 BGCs, respectively. A more in-depth analysis of these two strains performed by the combination of genome mining and methanolic extract characterization revealed that they could be able to produce molecules with antibiotic activities. In the same study, among *Actinobacteria*, the genome *Gordonia* sp. SL306 harbored the highest number of nonribosomal peptides (NRPs) related to BGCs. However, any retrieved BGC was linked to known secondary metabolites [23]. Finally, *Bacillus* sp. SL112 (among *Firmicutes*) hosted 14 BGCs in its genomes. Eight BGCs showed high similarities with already known BGCs retrieved in other *Bacillus* isolates and linked to the synthesis of some polyketides, nonribosomally synthesized peptides (such as amylocyclicin, bacillibactin, bacillaene, bacilysin, difficidin, fengycin, macrolactin, surfactin) with antibacterial, antifungal, and cytotoxic activities. The authors suggested that *Bacillus* sp. SL112 might be among those bacterial symbionts that are involved in the protection of their sponge host from predators and infections [23].

Among fourteen bacterial strains isolated from the freshwater sponge *Metania reticulata* (Bowerbank, 1863) from the Negro River (Brazil), Rozas et al. [27] individuated two bacterial strains (namely, MERETb.761 and MERETb.762) showing antimicrobial activity. Both bacterial strains inhibited the fungus *Aspergillus* sp., whereas MERETb.762 also inhibited *Staphylococcus aureus*. Two fractions of the MERETb.762 extract inhibited the degranulation of rat basophilic leukemia (RBL–_2_H_3_) cells and corresponded chemically to nitroaromatic compounds. The production of immunosuppressants is commonly considered a bacterial strategy to avoid expulsion from their hosts [45]. Rozas et al. [27] supposed that MERETb.762 might utilize such a strategy to maintain its relationship with the host. The authors also suggested that performing immunosuppressive assays on isolated compounds could enhance the discovery of novel bioactive molecules.

#### 3.2.2. Biosynthesis of Enzymes by Bacterial Isolates

Different enzyme activities were reported for unidentified bacterial isolates from the Lake Baikal sponges *L. baicalensis* and *B. bacillifera* [14]. Collagenase activity was in the range of 50–100% and 82–100% of bacteria isolated from *L. baicalensis* and *B. bacillifera*, respectively, followed by phosphatase (up to 90 and 64% of bacteria isolated from *L. baicalensis* and *B. bacillifera*, respectively) and phospholipase activities (e.g., 67% of *L. baicalensis* isolates). Caseinase activity was shown by 18 (*B. bacillifera* isolates) to 50% (*L. baicalensis* isolates) of the total tested strains. Lipase activity was detected in only 10–20% of bacterial strains isolates from both sponge species. According to the authors, based on these findings, sponge-associated bacteria, cleaving several organic compounds and thus supplying additional nutrients to their host, provide the sponge with food. 

#### 3.2.3. Polyketide Synthase Encoding Genes within the Associated Bacterial Communities

Polyketide synthases (PKS) are multifunctional enzymes responsible for the synthesis of low molecular weight bioactive metabolites. PKS sequences correspond to gene clusters in the microbial genomes. Therefore, the ability of microbial communities to produce bioactive molecules can be investigated by sequencing these genes. The amplification and subsequent sequencing of ketosynthase (KS) domain fragments of the PKS genes were used in the study of microbiomes of sponges *L. baicalensis* [46], *S. papyracea* [47], *Rezinkovia echinata* Efremova, 2004 [48], and *B. fungiformis* (Makuschok, 1927) [49]. In the first study, 15 PKS gene fragments, differing from each other by 35–65% by aminoacid sequences, were identified in the *L. baicalensis* microbial community [46] and related to *Alpha*-, *Beta*-, and *Deltaproteobacteria*, *Verrucomicrobia*, and *Cyanobacteria*. Some sequences were related to the genes involved in the biosynthesis of curacin A, stigmatellin, nostophycin, and cryptophycins. Interestingly, sequences showed a 50–82% with already known sequences. Later, the same authors identified 18 PKS gene fragments in the *S. papyracea* community metagenome [47]. The closest homologs belonged to the bacterial phyla *Cyanobacteria*, *Proteobacteria* (including *Betaproteobacteria*, *Deltaproteobacteria*, and *Gammaproteobacteria*), and *Acidobacteria*. The PKS gene spectrum significantly differed between *S. papyracea* and *L. baicalensis* [46]. Kaluzhnaya and Itskovich [48] identified a higher number (i.e., 36) of unique sequences of the PKS-gene fragments in the microbiome of *R. echinata*. Sequences were 57.3–99.6% identical to those of various taxonomic groups of bacteria: *Cyanobacteria* (*Scytonema* sp, *Crocosphaera watsonii*, *Cyanobium usitatum*, *Anabaena* sp., *Nostoc* sp., *Nostoc punctiforme*, *Trichormus variabilis*, *Synechococcus* sp.), *Verrucomicrobia* (*Opitutus terrae*, *Pedosphaera parvula*), *Betaproteobacteria* (*Piscinibacter aquaticus*), *Alphaproteobacteria* (*Bradyrhizobium* sp.), *Gammaproteobacteria* (Alteromonadaceae family), *Chloroflexi* (*Anaerolineae* family), *Firmicutes* (*Paenibacillus polymyxa*) and *Acidobacteria*. Notably, some of the discovered PKS genes were previously retrieved in the microbiomes of the sponge species *L. baicalensis* [46] and *S. papyracea* [47]. Finally, recently Kaluzhnaya and Itskovich [49] found that the *B. fungiformis*-associated microbial community included microorganisms (including the genera *Gemmatimonas*, *Rubrivivax,* and *Hydrogenophaga*) that could be potential producers of biologically active substances. The authors retrieved eight unique sequences, most of which were highly identical (97–99%) to the genes of PKS of prokaryotes from “core” communities (phyla *Cyanobacteria*, *Proteobacteria*, *Planctomycetes*, *Latescibacteria*, and *Gemmatimonadates*) associated with other Baikal sponge species (reported above), collected in different years and different parts of the lake [46,47,48].

Overall, these findings suggested the co-evolution of the host sponge and symbiotic microflora in the lake. On the other hand, several phylogenetically close PKS sequences were representative of Baikal planktonic microorganisms, indicating a close interaction between the symbiotic microbiome and the community of the environment surrounding the sponge host. Finally, the PKS sequences retrieved in Baikal sponges generally showed a low similarity with already known genes codifying for biologically active compounds, suggesting that freshwater sponge-associated bacteria might produce novel metabolites of a polyketide nature.

**Figure 3:**
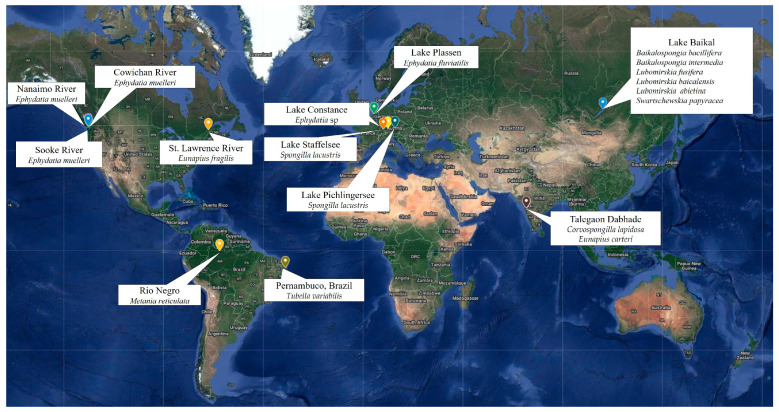
Freshwater sponges investigated in the field of associated prokaryotic communities and their geographical dislocation.

Figure 3 depicts the different freshwater sponge species investigated in the field of microbial ecology and shows the geographical dislocation of treated sponges.

## 4. Conclusions

Freshwater sponges are distributed globally at all latitudes and include more than 240 described species. They colonize continental habitats, spanning from the Arctic to tropical rain forests, that embrace both lotic and lentic systems. However, unlike their marine counterparts, understanding the roles played by sponge microbiomes in freshwater ecosystems is still in its infancy (e.g., their involvement in nutrient cycling and ecosystem functioning) and has yet to be clarified to disentangle the ecological and evolutionary significance of these unique and largely under-investigated microbial communities.

Overall, available data suggest that freshwater sponge symbionts may be able to utilize sponge-derived compounds and provide nutrients to the sponge host. Moreover, studies addressing the cultivable bacterial symbionts have highlighted their biotechnological potentialities as producers of bioactive compounds, suggesting their involvement in promoting sponge health. Both culture-dependent and culture-independent (only recently including next-generation sequencing) methods have been applied to describe the freshwater sponge-associated bacterial communities, which result generally predominated by *Proteobacteria*, *Bacteroidetes*, and *Actinobacteria*, with differences encountered with respect to marine sponges. However, fragmentary data are available on the composition of the prokaryotic communities associated with only a few different sponge species, which inhabit distant locations worldwide. Moreover, very diversified methodological approaches have been used, thus making poorly comparable the available data and arduous an exhaustive and solid assessment of the topic. Therefore, testing associated microbiome for biogeographic variation in the freshwater sponges could be useful to assess if sponge species-microbe relationships are conserved in freshwater systems as in the marine environment, and individuate relevant environmental factors, along with anthropogenic stressors (e.g., pollution), involved in the acquisition and structuring of their microbiomes. Moreover, most studies describing the sponge-associated prokaryotic communities (targeting quite exclusively the bacterial fraction) lack also considering the natural environment (i.e., water, sediment, and biofilm) surrounding the analyzed sponge individuals. This approach should be more extensively applied in the future to better discriminate between transient and truly associated bacteria.

Given the aforementioned, freshwater sponges may represent neglected hotspots of complex bacterial biodiversity with functional and metabolic features, as well as potential in the production of promising biotechnologically relevant bioactive compounds, that remain yet to be discovered.

## Figures and Tables

**Table 2:** Studies investigating the cultivable fraction of the freshwater sponge-associated bacterial communities.

**Sponge Species**	**Freshwater Sampling Site**	**Reference**
*Baikalospongia bacillifera* (Dybowski, 1880)	Lake Baikal (Russia)	[14]
*Baikalospongia intermedia* (Dybowsky, 1880)	Lake Baikal (Russia)	[14]
*Ephydatia* sp.	Lake Constance (Germany)	[41]
*Eunapius fragilis* (Leidy, 1851)	St. Lawrence River (North America)	[42]
*Lubomirskia fusifera* (Soukatschoff, 1895)	Lake Baikal (Russia)	[14]
*Lubomirskia baicalensis* (Pallas, 1776)	Lake Baikal (Russia)	[14]
*Spongilla lacustris* (Linnaeus, 1759)	Pichlinger See (Austria)	[23]
*Swartschewskia papyracea* (Dybowsky, 1880)	Lake Baikal (Russia)	[14]
*Tubella variabilis* (Bonetto and Ezcurra de Drago, 1973)	Artificial channel (Brazil)	[21]

**Table 3:** Bacterial isolates of biotechnological interest from freshwater sponges.

Sponge Species	Freshwater Sampling Site	Bacterial Isolate(s) ID	Activity	Reference
*B. bacillifera*	Lake Baikal	*Pseudomonas* strain 28Bb08	Detection of PKS genes	[43]
	Lake Baikal	Unidentified	Enzyme activity	[14]
	Lake Baikal	*Streptomyces* sp. IB2014/01-2; *Pseudonocardia* sp. IB2014/02-2	Antibiotic activities	[44]
*E. fluviatilis*	Vinkeveense Plassen Lake (The Netherlands)	*Pseudomonas* spp.	Antibiotic activities	[25]
*L. baicalensis*	Lake Baikal	Unidentified	Enzyme activity	[14]
*M. reticulata*	Negro River (Brazil)	*Bacillus* strain MERETb.762	Antibiotic activities	[27]
*S. lacustris*	Pichlinger See, Upper Austria	*Bacillus* sp. SL112	Antibiotic activities	[23]
	Pichlinger See, Upper Austria	*Gordonia* sp. SL306	Antibiotic activities	[23]
	Pichlinger See, Upper Austria	*Streptomyces* spp. SL203 and SL294	Antibiotic activities	[23]
*T. variabilis*	Artificial channel (Brazil)	Several genera	Antibiotic activities	[21]

## Data Availability

Data are available upon request.
